# Synthesis of Ca(OH)_2_ and Na_2_CO_3_ through anion exchange between CaCO_3_ and NaOH: effect of reaction temperature†

**DOI:** 10.1039/d2ra05827h

**Published:** 2022-11-11

**Authors:** Marco Simoni, Theodore Hanein, Chun Long Woo, Magnus Nyberg, Mark Tyrer, John L. Provis, Hajime Kinoshita

**Affiliations:** University of Sheffield, Department of Materials Science & Engineering Sheffield S1 3JD UK marco.simoni.w@gmail.com t.hanein@sheffield.ac.uk h.kinoshita@sheffield.ac.uk; CEMEX Asia Research AG Römerstrasse 13, Brügg 2555 Switzerland; Collegium Basilea Hochstrasse 51 Basel CH-4053 Switzerland

## Abstract

The CO_2_ released upon calcination of limestone accounts for the largest portion of the emissions from the cement, lime, and slaked lime manufacturing industries. Our previous works highlighted the possibility for a no-combustion decarbonisation of CaCO_3_ through reaction with NaOH solutions to produce Ca(OH)_2_ at ambient conditions, while sequestrating the process CO_2_ in a stable mineral Na_2_CO_3_·H_2_O/Na_2_CO_3_. In this study, the effect of temperature was assessed within the range of 45–80 °C, suggesting that the process is robust and only slightly sensitive to temperature fluctuations. The proportioning of the precipitated phases Na_2_CO_3_·H_2_O/Na_2_CO_3_ was also assessed at increasing NaOH molalities and temperatures, with the activity of water playing a crucial role in phase equilibrium. The activation energy (*E*_a_) of different CaCO_3_ : NaOH : H_2_O systems was assessed between 7.8 kJ·mol^−1^ and 32.1 kJ·mol^−1^, which is much lower than the conventional calcination route. A preliminary energy balance revealed that the chemical decarbonisation route might be ∼4 times less intensive with respect to the thermal one. The present work offers a further understanding of the effect of temperature on the process with the potential to minimise the emissions from several energy-intensive manufacturing processes, and correctly assess eventual industrial applicability.

## Abstract 2

The chemical decarbonisation of CaCO_3_ was successfully tested in a range of mild temperatures simulating those oscillations occurring within processes, and the kinetic parameters were gained.

## Graphical abstract

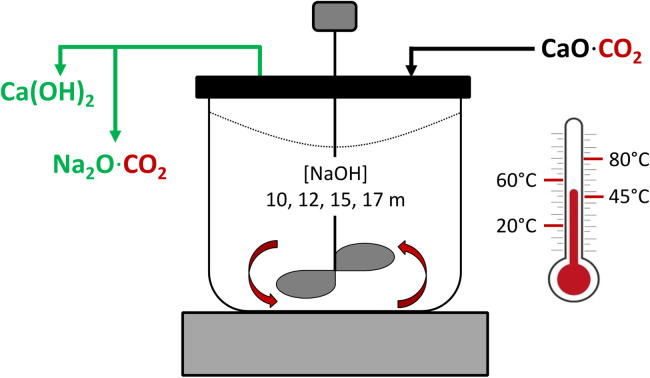

## 1 Introduction

The calcination of limestone to give lime (CaCO_3_ → CaO + CO_2_) is a crucial step for the production of Portland cement (PC), lime (CaO), slaked lime (Ca(OH)_2_), and soda ash (Na_2_CO_3_), whose worldwide markets exceed 4 Gt,^1^ ∼55 Mt, ∼15 Mt,^2^ and 50 Mt ^3^ per year, respectively. Given the heavy carbon footprint of such calcination step,^4^ these industrial realities are responsible for a significant portion of the total CO_2_ emissions worldwide.^1,5,6^ Depending on the targeted product, the very energy-intensive^7^ calcination of limestone might occur in different calcination designs, with varying efficiencies and carbon emissions;^8^ specifically, 0.8, 1.0–1.8, and 0.4 t CO_2_ are emitted to produce 1 t of cement, lime/slaked lime, and soda ash, respectively. However, independently from the calcination unit considered, the emissions arising from this step arise from both the fuel-derived and process CO_2_. While the process CO_2_ results from the stoichiometry of CaCO_3_ decomposition, the fuel-derived emissions arise from combustion of fuels (primarily fossil fuels) necessary to achieve the calcination temperature (above 900 °C).^9^ The achievement of those environmental goals set by the UN in 2015 (at least 80% of CO_2_ emission cut from the industry sector by 2050^10^) requires a significant contribution from the cement, lime, and soda ash industries. To achieve this, the application of carbon capture and storage (CCS) technologies^11^ and the use of alternative fuels^12^ are expected to be the best candidates; however, both these solutions assume that the thermal calcination of limestone (and the process CO_2_) is unavoidable. In contrast to this, recent investigations^13–16^ highlighted the possibility for alternative decarbonisation routes which apply no-combustion approaches for the synthesis of the essential chemical CaO^17^ from CaCO_3_. As far as we are aware, the chemical decarbonisation route here characterised represents the only alternative for the sustainable co-synthesis of Ca(OH)_2_ and Na_2_CO_3_. The stoichiometry of the key-reaction is reported in eqn (1); it occurs at ambient conditions and implies the reaction between CaCO_3_ and aqueous NaOH solutions.

1 CaCO_3_ + 2NaOH + *x*H_2_O → Ca(OH)_2_ + Na_2_CO_3_·*x*H_2_O

The process is relatively simple; however, the full fundamental understanding of the system is necessary to determine the feasibility of any scaled-up industrial process, and the reaction rate is assessed in this work. The reaction rate may be defined through eqn (2),^18^ where the variables *t*, *T*, and *α* (with 0 < *α* < 1) represent the reaction time, temperature, and extent of reaction, respectively.^18^ The function *f*(*α*) represents the kinetic reaction model depending on the mechanism assessed,^19^ while *f*(*T*) reflects the Arrhenius equation (eqn (3)), depending on the temperature (*T*), the activation energy (*E*_a_), and the pre-exponential factor (*A*).

2 ∂*α*/∂*t* = *f*(*T*) × *f*(*α*)

3
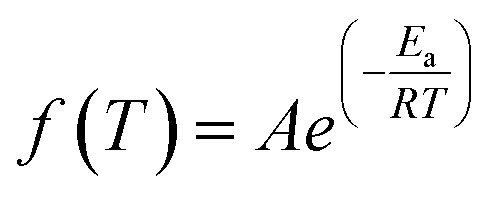


Despite the mild temperature range here considered (45–80 °C), it is crucial to assess the response of a given process to eventual fluctuations; ideally, a robust route would not be significantly affected by these variations in the processing parameters. The title study aims to gain a deeper insight into the behaviour of the system considered at increasing temperatures, therefore providing essential information for any eventual scale-up.

## 2 Materials and methods

### 2.1 Experimental procedure

The materials used in this work are reagent grade chemicals: Sigma-Aldrich CaCO_3_ (≥99%), Honeywell Fluka NaOH (≥97%), and distilled water. The ternary composition (wt%) 26.9 (CaCO_3_) : 32.7 (NaOH) : 40.4 (H_2_O) was first investigated, allowing for a 0.86 conversion efficiency (*α*) in a previous study;^16^ therefore, a 20 M NaOH (mol kg_H_2_O_^−1^) was first considered. The relatively high water to solids ratio (1.5 g g^−1^) ensured homogeneous mixing of the solid powders even at milder stirring conditions, as described below, with respect to a previous characterisation.^14^

**Fig. 1:**
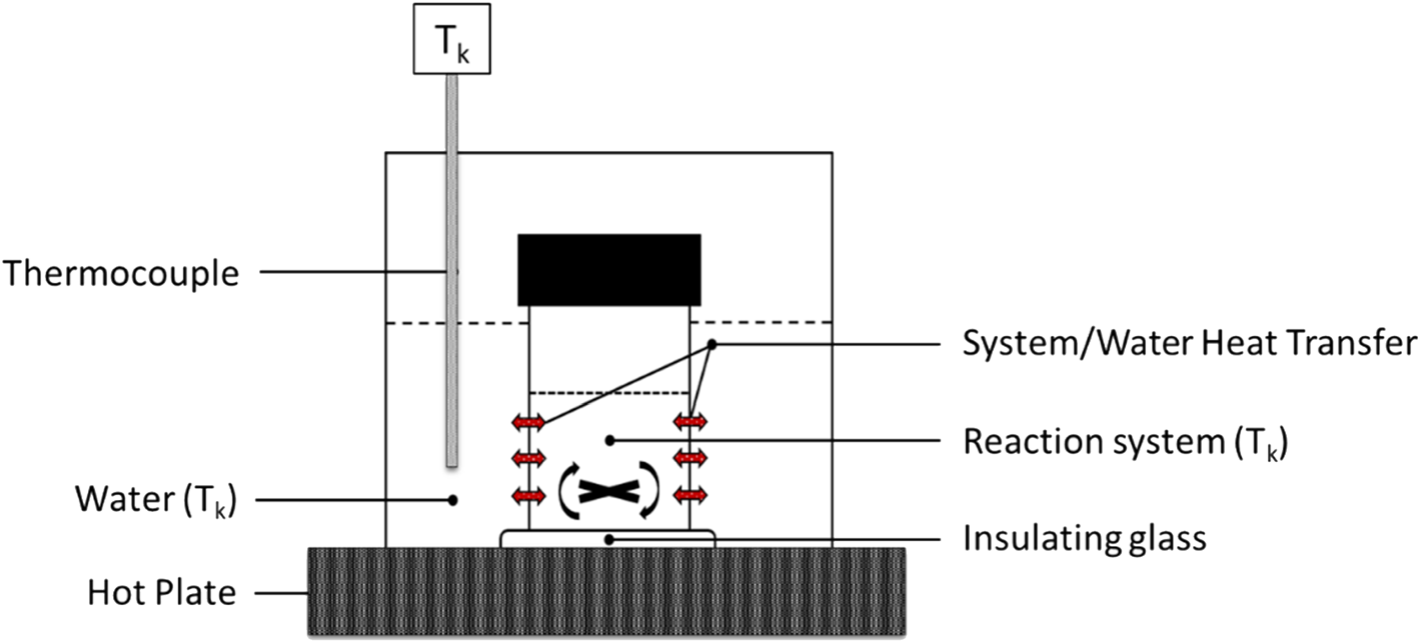
Illustration of the experimental set up discussed above, highlighting the indirect temperature control on the reaction system by setting the surrounding water environment at a *T*_k_ temperature through external thermocouple.

The experimental set up is illustrated in Fig. 1. The 20 m NaOH solutions were prepared in a polypropylene plastic vessel, and placed in the pre-heated water bath for 30 min at the target temperatures *T*_k_ of 45 °C, 60 °C, or 80 °C.

Direct contact between the hot plate and the bottom of the reaction vessel was avoided through the placement of an insulating glass disk. The CaCO_3_ powders were also pre-heated at 80 °C for 2 hours to remove excess water. The CaCO_3_ was then quickly added to the NaOH solutions and manually mixed, at first, to prevent the agglomeration of the solids. Subsequently, the vessel was covered with a lid to minimise the loss of water and heat throughout the reaction; a magnetic stirring of 300 rpm was set up at increasing residence times of 1, 2, 3, 4 and 5 min. Longer residence times were not considered, since our previous study^14^ highlighted that negligible reaction progression would occur beyond 5 min. The reaction was also performed in non-isothermal conditions, upon initial heating of the NaOH solutions at the targeted temperatures *T*_i_ of 45 °C, 60 °C, and 80 °C (to be referred as 45 °C_5 min_RT, 60 °C_5 min_RT, and 80 °C_5 min_RT, respectively). A residence time of 5 min was solely considered to produce these samples.

Upon reaction, all the pastes were mixed with methanol (30 mL) for further 5 min, ensuring the removal of the unreacted NaOH, whose solubility in methanol is 238 g L^−1^;^20^ whereas, the negligible solubility of CaCO_3_,^21^ Ca(OH)_2_,^22^ Na_2_CO_3_ (ref. 23) and Na_2_CO_3_·H_2_O^23^ in methanol would ensure an unmodified solid phase assemblage upon washing. The solid products and the liquid streams could then be collected separately using Buchner filtration. The solids were dried at 80 °C for 2 h, before being manually ground and sieved below 63 μm for subsequent characterisation though thermogravimetry and X-ray diffraction.

An additional set of experiments was conducted to estimate the *E*_a_ of the decarbonisation reaction at different ternary compositions (Table 1), selected from our previous study.^16^ The same procedure described above was applied here, with the solids being reacted for 1 min at constant temperatures *T*_k_ of 30 °C, 45 °C, 60 °C and 70 °C. The datasets used and/or analysed during the current study are available from the corresponding author on reasonable request.

**Table 1:** Starting compositions (wt%) and the molar ratio NaOH/CaCO_3_ of the samples. The parameter labelled as α reflects the conversion extent values (0 < *α* < 1) of CaCO_3_ into Ca(OH)_2_ reported in our previous study

ID	NaOH (wt%)	CaCO_3_ (wt%)	H_2_O (wt%)	NaOH/CaCO_3_ (mol mol^−1^)	H_2_O/CaCO_3_ (g g^−1^)	H_2_O/NaOH (mol mol^−1^)	α^16^
NaOH_10m	14.3	14.3	71.4	2.5	3.0	5.5	0.07
NaOH_12m	24.5	24.5	51.0	2.5	2.1	4.6	0.46
NaOH_15m	30.1	20.0	49.9	3.8	2.5	3.7	0.69
NaOH_17m	37.2	8.1	54.7	11.5	6.7	3.3	0.96

### 2.2 Characterisation techniques

#### 2.2.1 X-ray diffraction (XRD)

The reaction products were qualitatively identified by X-ray diffraction (XRD) and following analysis on the Highscore-Plus software with the PDF^−4^ 2019 database. The measurements were performed using a Bruker D2 PHASER desktop X-ray diffractometer in the Bragg–Brentano geometry equipped with a CuKα radiation source running at 30 kV and 10 mA. The instrument is equipped with a one-dimensional LYNXEYE detector and a 1 mm divergence slit. All samples were in a powder form, and they were loaded onto the sample holder with 2.5 cm diameter and 1 mm deep. All the analyses were conducted between 5° and 80° (2*θ*) with a step size of 0.02° at 0.5 s per step, with the stage rotating at 15 rpm to improve counting statistics.

#### 2.2.2 Thermogravimetry (TG/DTG)

**Fig. 2:**
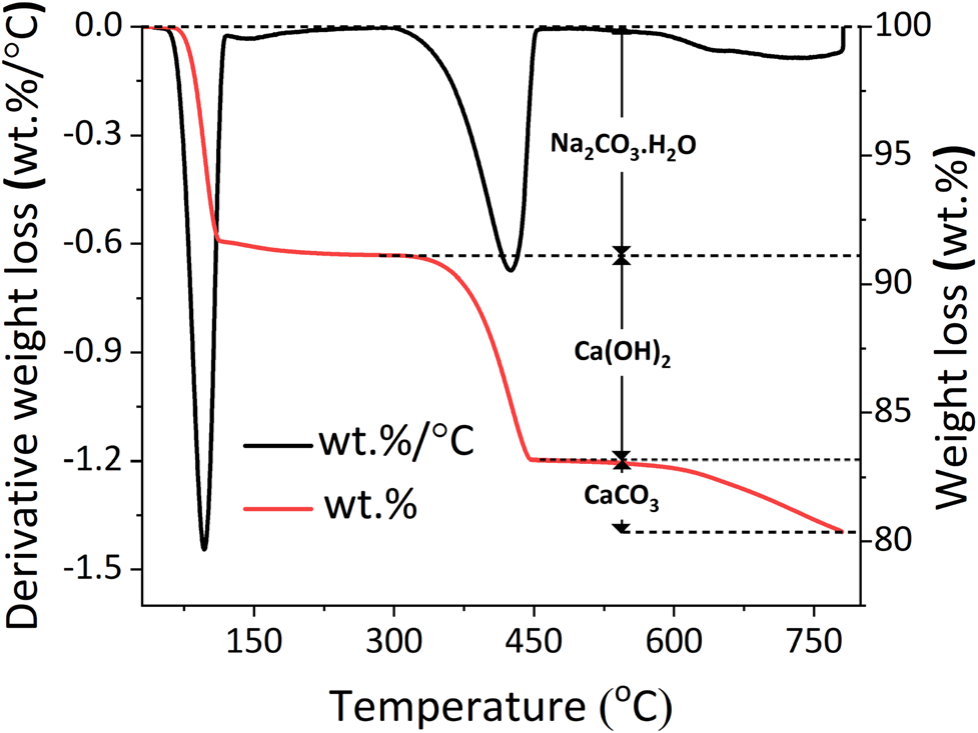
Generic temperature-trend of the TG data (wt%) and DTG data (wt%/⁰C), red and black lines, respectively, showing the weight loss events of the sample associated with: Na_2_CO_3_·H_2_O, Ca(OH)_2_, and CaCO_3_.

A PerkinElmer TGA 4000 was used to provide thermogravimetric analysis (TGA) for the reaction products; approximately 40 mg of sample were subjected to a temperature ramp from 30 °C to 800 °C at the heating rate of 10 °C min^−1^, with a 40 mL min^−1^ of N_2_ flow. The heating program was set up to held the sample at 800 °C for 1 hour to ensure complete loss of CO_2_ from CaCO_3_; the analysis temperature was not exceeding 800 °C to avoid the melting or weight loss from Na_2_CO_3_. To identify evolving gases, a Hiden mass spectrometer (HPR-20 GIC EGA) was used to record the signals for CO_2_ and H_2_O. An example of TG analysis is presented in Fig. 2, where the mass loss events are linked to Ca(OH)_2_, Na_2_CO_3_·H_2_O and CaCO_3_. Together with the weight loss, the DTG trend is also reported to ensure the distinction of eventual overlapping signals. The extent of reaction was calculated based on the amount of Ca(OH)_2_ (ref ^24^) and unreacted CaCO_3_,^25^ based on the respective thermal decompositions. The content of Na_2_CO_3_·H_2_O could similarly be estimated in the temperature range 50–130 °C.^26^

The measurement was repeated 6 times for each sample to estimate the measurement error (±0.16 wt%, ± 0.10 wt% and ± 0.16 wt% for Na_2_CO_3_·H_2_O, Ca(OH)_2_ and CaCO_3_, respectively).5
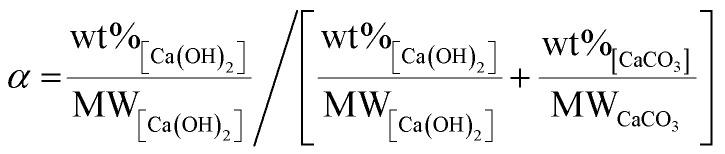


## 3 Theory

This investigation aims to provide the scientific community with a deeper insight into the chemistry and energetic demand of a novel CaCO_3_ decarbonisation route,^16^ with the potential to lead the transition towards sustainable cement, lime, and soda ash industries. To highlight this, an energetic comparison with the state-of-the-art processes is presented in Section 5, highlighting the potential advantages of the alternative one. To do that, the concomitant by-production of soda ash Na_2_CO_3_ was also considered, whose conventional manufacturing route through Solvay process requires a high energy supply. This early-stage investigation, together with other ones focusing on the processing conditions^14^ and the nature of the raw materials used,^15^ will provide a strong base to the potential scale-up of the process. In these terms, future work will aim to design a laboratory-scale process simulating the ideal industrial one, providing a stronger dataset to assess the effective energy requirements of the route, and therefore allowing for a proper techno-economic analysis.

## 4 Results and discussion

### 4.1 Effect of temperature and CO_2_ capture

**Fig. 3:**
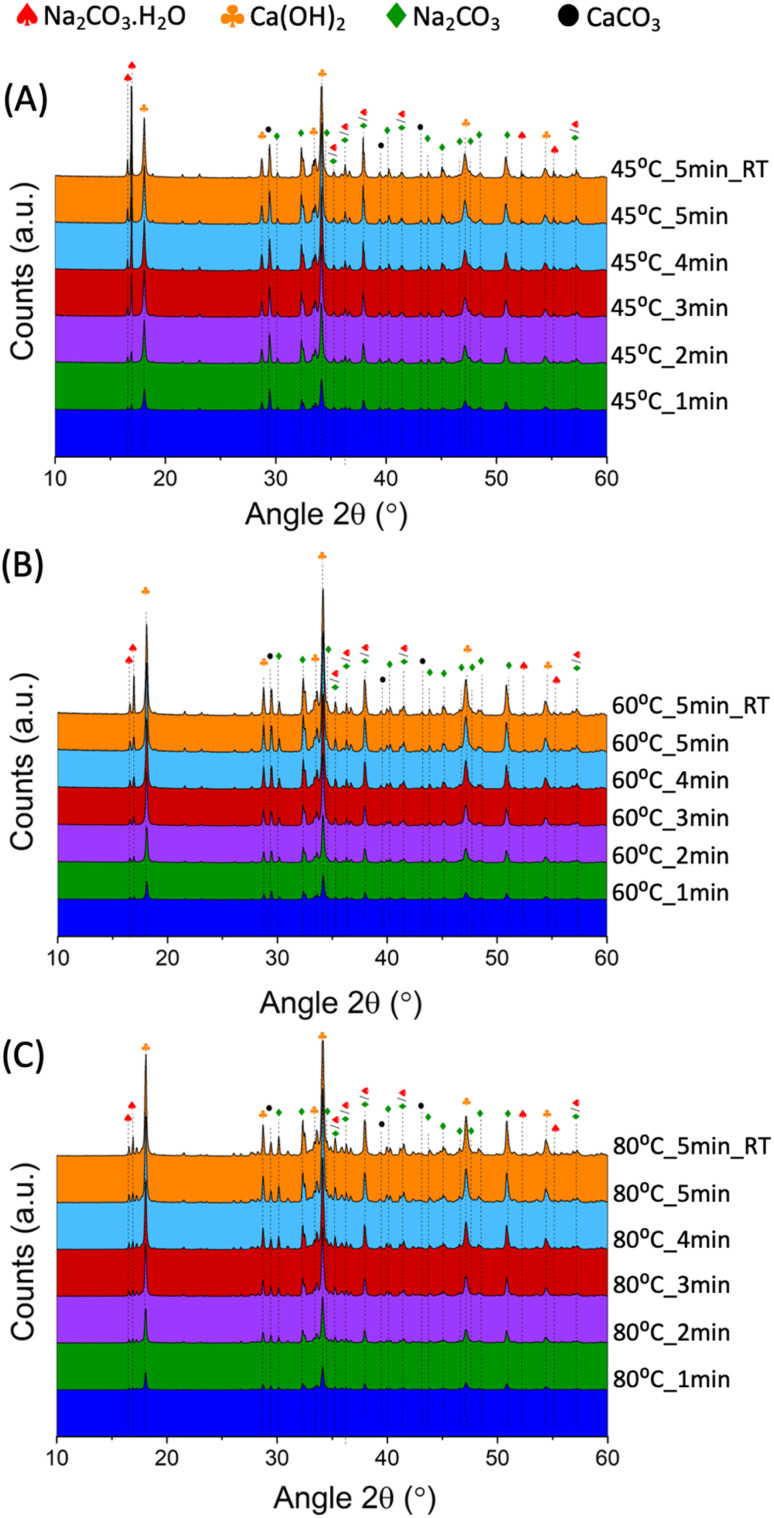
XRD patterns for the samples produced at a constant temperature of 45 °C (A), 60 °C (B), and 80 °C (C), and increasing residence times; the patterns also include those samples produced at ambient conditions and with an initial temperature of 45 °C, 60 °C, and 80 °C.

The XRD analysis of the 45 °C_n, 60 °C_n, and 80 °C_n samples are shown in Fig. 3A–C, respectively.

**Fig. 4:**
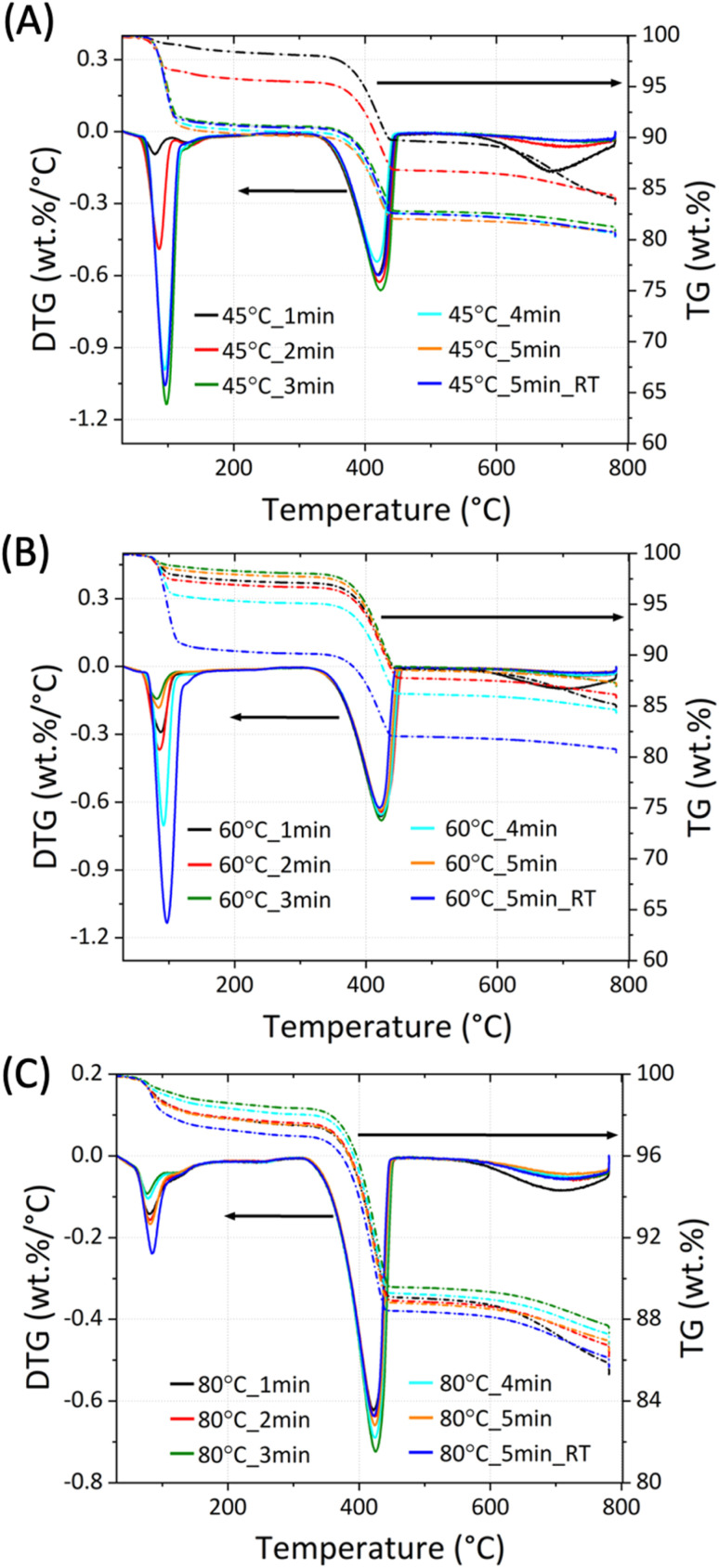
TG/dTG analysis for the samples series 45 °C_n (A), 60 °C_n (B), and 80 °C_n (C) showing the weight losses in the temperature ranges of 50–130 °C, 310–470 °C and 560–800 °C corresponding to the presence of Na_2_CO_3_·H_2_O, Ca(OH)_2_ and CaCO_3_, respectively.

Upon reaction, only Ca(OH)_2_ (ICSD collection code: #191851), Na_2_CO_3_ (ICSD collection code: #5009), Na_2_CO_3_·H_2_O (ICSD collection code: #1852), and unreacted CaCO_3_ (ICSD collection code: #80869) could be detected, with respective main reflection angles at 2*θ* of 29.5°,^27^ 34.1°,^28^ 16.9° (ref. 29) and 30.1°.^30^ No additional phases were detected, suggesting the absence of secondary and competing reactions. The TG data for the 45 °C_n, 60 °C_n and 80 °C_n series is shown in Fig. 4A–C, respectively.

The phase quantification was performed through the weight losses observed in the TG data for CaCO_3_ (at 560–800 °C), Ca(OH)_2_ (at 310–470 °C) and Na_2_CO_3_·H_2_O (at 50–130 °C). The estimated quantities for all the samples discussed are summarised in Table 2, where the processing conditions used are also reported.

**Table 2:** Processing conditions and phases composition (wt%) gained from thermogravimetric analysis, together with the Loss On Ignition (LOI) expressed in % and the Na_2_CO_3_·H_2_O/Ca(OH)_2_ molar ratio registered for all the samples discussed

Sample ID	CaCO_3_ (wt%)	Ca(OH)_2_ (wt%)	Na_2_CO_3_·H_2_O (wt%)	Na_2_CO_3_ (wt%)	LOI (%)	Na_2_CO_3_·*x*H_2_O/Ca(OH)_2_ (mol%/mol%)
45 °C_1 min	13.7	34.5	6.6	45.2	15.4	1.1
45 °C_2 min	6.4	35.6	23.6	34.4	14.9	1.1
45 °C_3 min	4.2	34.3	55.9	5.6	18.3	1.1
45 °C_4 min	4.8	32.9	59.6	2.7	18.8	1.1
45 °C_5 min	3.2	33.5	61.6	1.7	18.5	1.1
45 °C_RT_5 min	4.6	34.7	57.0	3.7	18.7	1.1
60 °C_1 min	8.9	34.8	15.1	41.2	14.6	1.1
60 °C_2 min	4.5	36.8	18.0	40.7	13.5	1.1
60 °C_3 min	4.2	37.7	8.6	49.5	12.2	1.1
60 °C_4 min	4.3	36.7	8.9	50.1	15.0	1.1
60 °C_5 min	3.1	37.8	11.1	48.0	12.2	1.1
60 °C_RT_5 min	3.2	33.5	61.6	1.7	18.5	1.1
80 °C_1 min	8.5	34.7	11.1	45.7	13.8	1.2
80 °C_2 min	6.1	35.9	11.0	47.0	13.0	1.2
80 °C_3 min	5.2	36.1	6.6	52.1	12.0	1.2
80 °C_4 min	5.5	36.1	7.9	50.5	12.3	1.2
80 °C_5 min	5.2	35.8	11.2	47.8	12.6	1.2
80 °C_RT_5 min	6.0	35.2	15.0	43.8	13.4	1.2

It must be mentioned that the content of Na_2_CO_3_ was calculated by subtracting the sum of the other quantified phases Ca(OH)_2_, CaCO_3_ and Na_2_CO_3_·H_2_O from the total mass (100%). In fact, since Na_2_CO_3_ would start decomposing above 851 °C,^31^ it could not be directly quantified by TG analysis (up to 800 °C) through the detection of the relevant peak. A higher content of Na_2_CO_3_ could also be suggested by the lower LOI registered for samples with similar reaction efficiencies (Table 2). This aspect will be extensively discussed in Section 3.2. However, the quantification was considered reliable since the XRD analysis confirmed the absence of any additional phases in the solid products. Moreover, the ratio between the precipitated Na_2_CO_3_·H_2_O/Na_2_CO_3_ and Ca(OH)_2_ (mol%/mol%) revealed a good accordance to the stoichiometry expressed in eqn (1) (Table 2). In fact, 1 mol of both Ca(OH)_2_ and Na_2_CO_3_·H_2_O/Na_2_CO_3_ should precipitate for each mol of CaCO_3_ reacted, and the resulting molar ratio between Na_2_CO_3_·H_2_O/Na_2_CO_3_ and Ca(OH)_2_ was expected to be close to unity. Specifically, the ratios were suggesting a slight over-precipitation of Na_2_CO_3_·H_2_O and Na_2_CO_3_ with respect to Ca(OH)_2_ for all the systems studied and that could possibly be reflecting the distribution of the positive (Ca^2+^) and negative (CO_3_^2−^) charged sites on the surface of the CaCO_3_. Statistically, a 27% excess of negatively charged sites may be found on the CaCO_3_ surface,^32^ justifying the greater affinity of the CaCO_3_ to interact with the cationic species Na^+^ in the liquid bulk to form Na_2_CO_3_·H_2_O/Na_2_CO_3_.

**Fig. 5:**
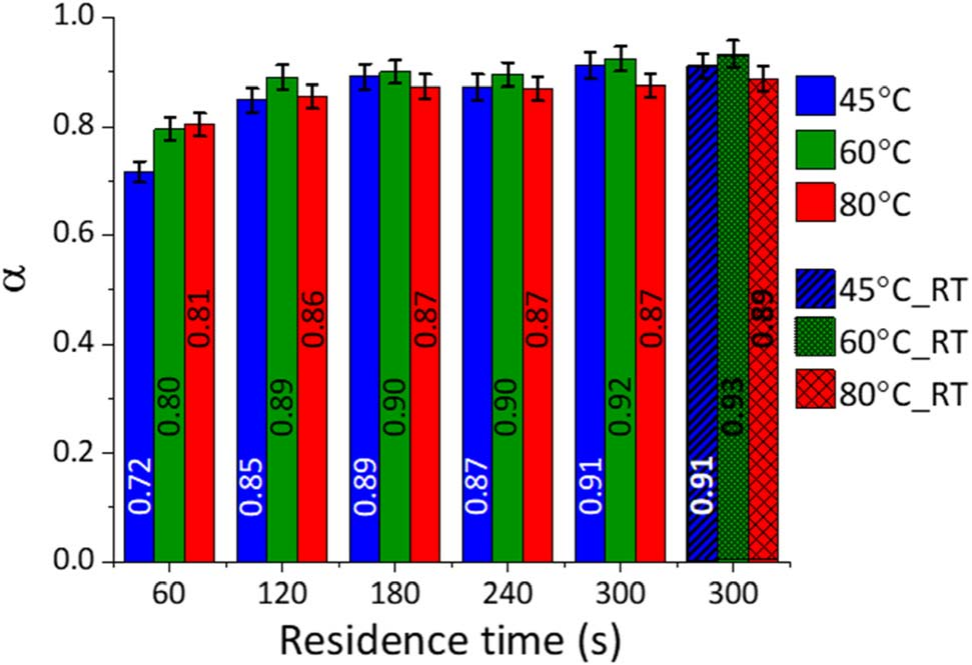
Extent of reaction (*α*) for the samples at different temperatures (45, 60 and 80 °C)and increasing residence times, based on TG analysis; the outcomes are also reported for those samples produced without actively maintaining the temperature throughout the reaction.

Based on the TG data, the extent of reaction (*α*) was calculated for each system, and the outcomes are reported in Fig. 5.

The conversion of CaCO_3_ was high (0.7 < α < 0.8) in the tested reaction conditions, in line with the qualitative XRD data (Fig. 3A–C), showing progressive decrease in the intensity of the CaCO_3_ main peak at 29.5° 2*θ*. During the first minute, the system temperature had a significant impact on the extent of the reaction; enhanced conversion efficiencies were gained at higher temperatures, while limited effects were observed at longer residence times. It seems likely that the higher conversion registered at short residence times and higher temperatures could be linked to the lower viscosity of the NaOH solutions, favouring the ionic mobility and the enhanced interaction between the dissolved species and the solid surface and bulk.

**Fig. 6:**
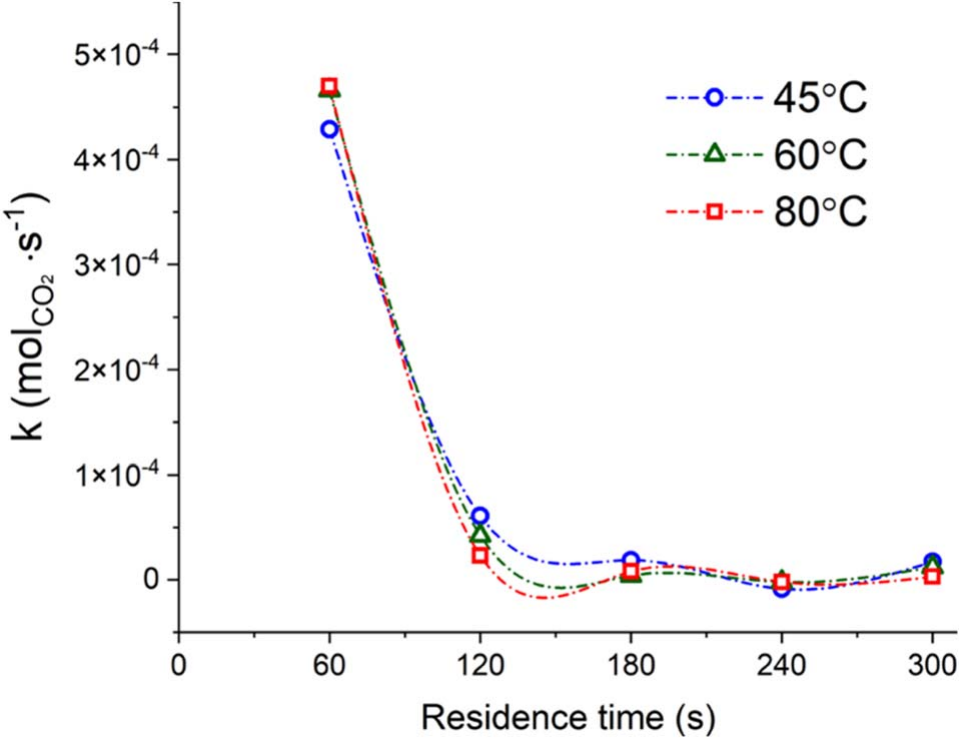
CO_2_ capture rate, expressed in moles of CO_2_ precipitated as Na_2_CO_3_·*x*H_2_O per second of reaction progression, for all the residence times considered at constant temperature; the samples reacted at ambient conditions were not considered for this graph. The lines only work as a guide for the eye.

The efficiency of the system may also be expressed in terms of CO_2_ capture, expressed as moles of CO_2_ precipitated as Na_2_CO_3_·*x*H_2_O per second of reaction progression. As reported in Fig. 6, the CO_2_ capture rate was decreasing from ∼4.5 × 10^−4^ mol sec^−1^ of CO_2_ in the first minute of reaction down to two orders of magnitude below (∼10^−6^ mol sec^−1^ of CO_2_) after 5 min of contact time. In other terms, around the 80% of the total process CO_2_ initially introduced was effectively captured after 1 min of reaction.

The samples reacted at ambient conditions (45 °C_5 min_RT, 60 °C_5 min_RT, and 80 °C_5 min_RT) indicated extent of reactions like those reacted at a constant temperature after 5 min (Fig. 5). Decreasing temperature trends could be detected for 45 °C_5 min_RT, 60 °C_5 min_RT, and 80 °C_5 min_RT, with final temperatures of 21.5, 42.1, and 53.6 °C, respectively, after 5 min of residence time. Evidently, the temperature of the system was not significantly influencing the progression of the reaction for a residence time of 5 min.

### 4.2 Partitioning of Na_2_CO_3_·H_2_O and Na_2_CO_3_

**Fig. 7:**
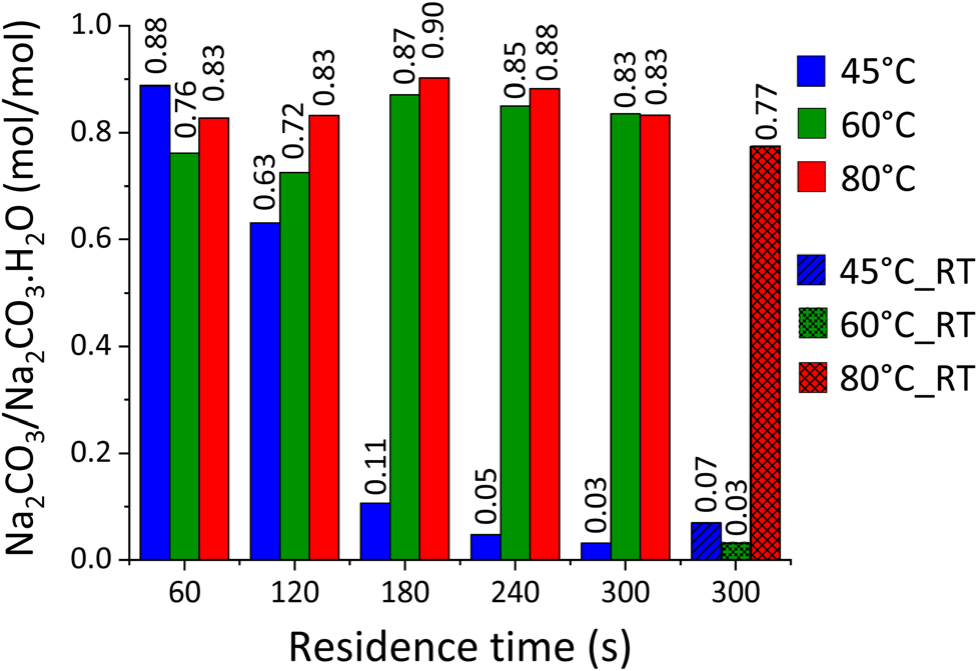
Molar fraction *ν* of Na_2_CO_3_, expressed as molar ratio between Na_2_CO_3_ and Na_2_CO_3_·H_2_O, for the samples produced at 45, 60 and 80 °C at increasing residence times.

The reaction at different conditions resulted in the sequestration of the process CO_2_ through precipitation of Na_2_CO_3_·H_2_O and Na_2_CO_3_ in different proportion, with the *x* value (eqn (1)) equal to 1 and 0, respectively. Based on the data obtained from TGA (Fig. 4A–C), the molar fraction *ν* of precipitated Na_2_CO_3_, with 0 < *ν* < 1, was calculated by dividing the moles of Na_2_CO_3_ in the samples by the total moles of Na_2_CO_3_·H_2_O and Na_2_CO_3_. As introduced in Section 2.1, most samples were produced at a constant temperature (*T*_k_ = 45 °C, 60 °C, and 80 °C) whereas others were only initially set up at targeted temperature values (*T*_i_ = 45 °C, 60 °C, and 80 °C), following which the system was left at ambient conditions and was allowed to achieve Tf (with *T*_f_ < *T*_i_). As reported in Fig. 7, the precipitation of Na_2_CO_3_ was dominant at *T*_k_ = 45 °C up to 2 min of reaction; beyond that, the precipitation of Na_2_CO_3_·H_2_O was larger despite the constant temperature. Whereas, when considering the systems at *T*_k_ = 60 °C and *T*_k_ = 80 °C, the precipitation of Na_2_CO_3_ was dominant for all the residence times investigated.

Different outcomes were observed when considering the samples reacted at ambient conditions, suggesting a key-role of the temperature on the equilibrium between Na_2_CO_3_·H_2_O and Na_2_CO_3_. In fact, while Na_2_CO_3_·H_2_O was dominant at *T*_i_ = 45 °C and *T*_i_ = 60 °C, with respective *T*_f_ values of 21.5 °C and 42.1 °C, Na_2_CO_3_ was the main species precipitating at *T*_i_ = 80° (*T*_f_ = 53.5 °C). Likely, such a different proportion gained at different values of *T*_i_ and *T*_f_ might be explained by considering the standard enthalpy of reaction Δ*H*_R_ for *x* = 0, 1 in eqn (1). The calculation was performed by application of the Hess law and considering standard enthalpies of formation of −1207.4, −426.7, −285.8, −986.1, −1429.7, and −1129.2 kJ mol^−1^ for CaCO_3_,^33^ NaOH,^34^ H_2_O,^34^ Ca(OH)_2_,^34^ Na_2_CO_3_·H_2_O,^33^ and Na_2_CO_3_,^33^ respectively. It appears that the precipitation of Na_2_CO_3_·H_2_O is enhanced at lower temperatures (Δ*H*_R_ = −69.2 kJ mol^−1^) than that of Na_2_CO_3_ (Δ*H*_R_ = −54.5 kJ mol^−1^), justifying the results just discussed. The XRD analysis (Fig. 3A–C) was qualitatively in accordance with the quantification of Na_2_CO_3_·H_2_O and Na_2_CO_3_ gained from TGA for both the samples reacted at constant and varying temperatures. In fact, low contents of Na_2_CO_3_·H_2_O (main peak at 16.9° 2*θ*) were suggested for all the samples reacted at *T*_k_ = 80 °C, whereas increased intensities were observed for the *T*_k_ = 45 °C series above 2 min of residence time and the sample reacted at ambient conditions with *T*_i_ = 60 °C.

**Fig. 8:**
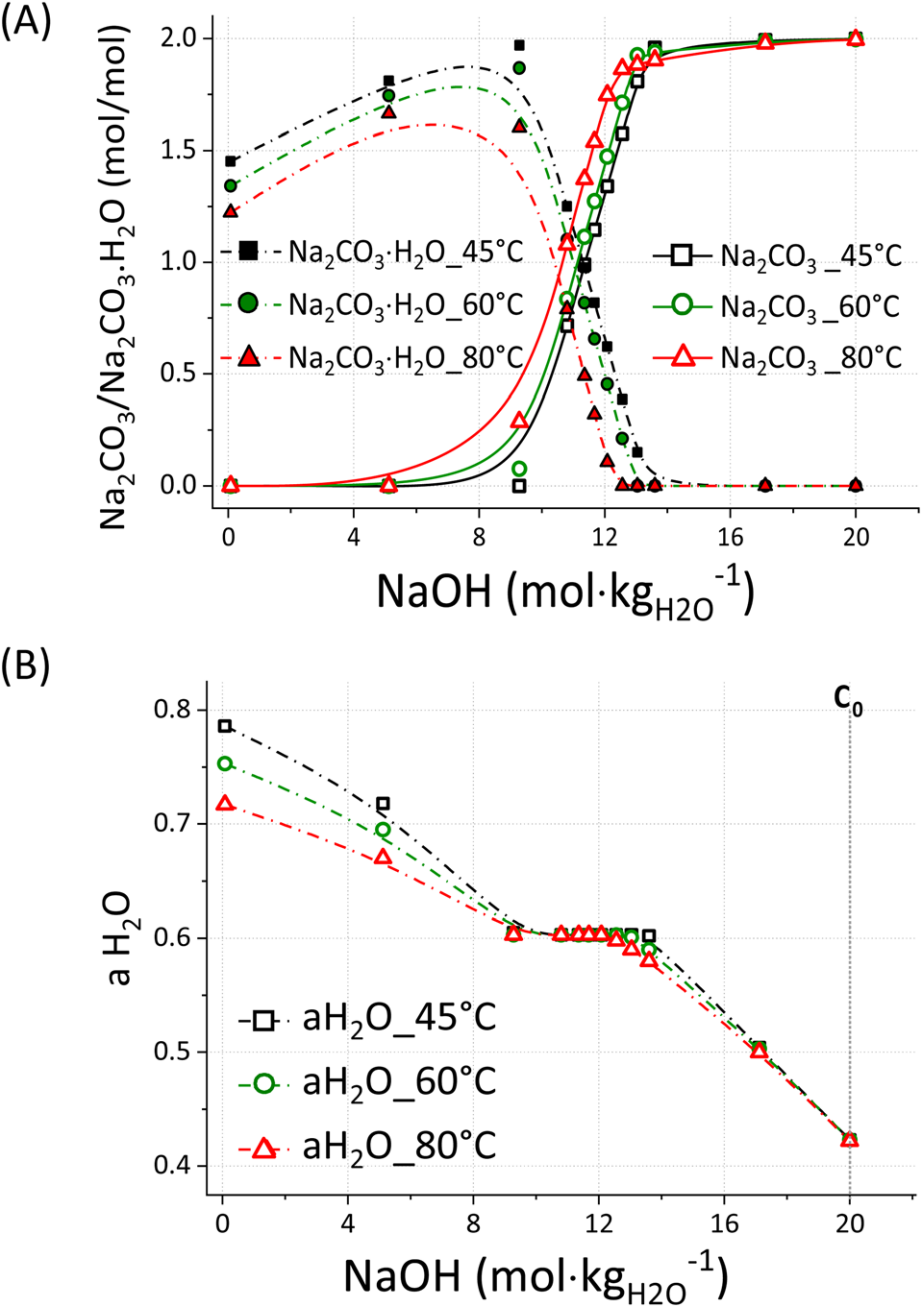
PHREEQC simulation showing the molar ratio between the precipitated Na_2_CO_3_ and Na_2_CaO_3_·H_2_O (A) and the activity of water aH_2_O (B) at 45, 60 and 80 °C and increasing NaOH molalities, up to 20 m; the concentration *C*_0_ (B) indicates the initial NaOH concentration considered for the samples here discussed.

The equilibrium between Na_2_CO_3_·H_2_O and Na_2_CO_3_ was studied by performing targeted simulations of a simplified system using the PHREEQC software^35^ with the PITZER_(2018)_ database; an overview of the outcomes is reported in Fig. 8A and B.

The simulations were conducted for a system composed of 1 mole of Na_2_CO_3_·H_2_O and 1 mole of Na_2_CO_3_, both dissolved in 0.1 kg of water at *T*_k_ = 45 °C, *T*_k_ = 60 °C, and *T*_k_ = 80 °C, with increasing concentrations of NaOH up to 20 m. The simulated system does not contain Ca^2+^ ions as in the experimentally tested systems but it is useful to understand the general behaviour of Na_2_CO_3_·H_2_O and Na_2_CO_3_ in highly concentrated NaOH solutions. As reported in Fig. 8A, Na_2_CO_3_·H_2_O and Na_2_CO_3_ are the dominant species at [NaOH] < 10 m and [NaOH] > 12.5 m, respectively, while the co-precipitation of both species occurs at 10 m < [NaOH] < 12.5 m. Such behaviour might likely depend on the activity of water, which lowers at increasing solute concentrations and system temperature.^36^ Given the higher proportion of Na_2_CO_3_ at *T*_k_ = 60 °C and *T*_k_ = 80 °C with respect to *T*_k_ = 45 °C, it is likely that such phase is favoured at a lower activity of water, *i.e.* higher temperature. However, an unexpected trend is shown in Fig. 7 for the samples reacted at *T*_k_ = 45 °C; in fact, Na_2_CO_3_ was the main phase up to 2 min of reaction, whereas the content of Na_2_CO_3_·H_2_O sharply increased beyond that. Likely, the consumption of alkalinity related to the progression of eqn (1) resulted in a lower activity of water, allowing for the precipitation of Na_2_CO_3_·H_2_O rather than Na_2_CO_3_. In fact, a higher conversion extent (α = 0.89) was registered after 3 min of reaction, if compared with the outcome after 1 min (α = 0.72) and 2 min (α = 0.85). Most likely, a low activity of water could be maintained at *T*_k_ = 60 °C and *T*_k_ = 80 °C, even upon enhanced reaction progression, *i.e.* alkalinity consumption. Whereas the alkalinity drop occurring at an enhanced conversion extent was enough to increase the activity of water in those systems reacted at *T*_k_ = 45 °C. This is also confirmed by the experiments conducted at ambient conditions, since the system at *T*_i_ = 60 °C mostly formed Na_2_CO_3_·H_2_O at the end of the reaction, when *T*_f_ = 42.1 °C was registered.

A deeper insight allows for the explanation of the dynamic situation occurring at 10 m < [NaOH] < 12.5 m; in such interval, the system achieves a transitionary aH_2_O value of 0.603. That corresponds to a chemical potential *u* of −241.3 kJ mol^−1^ (eqn (6)), where *u*_0_ is the standard chemical potential of formation of pure water,^37^*R* the gas constant, and *T* the temperature in K.

6 *u* = *u*_0_ + *RT* ln *a*_w_

At this point, the transition Na_2_CO_3_·H_2_O/Na_2_CO_3_ is swapped (Fig. 8A), promoting the precipitation of Na_2_CO_3_·H_2_O and Na_2_CO_3_ above and below an activity of water of 0.603, respectively. To explain the constant water activity value of 0.603, the gradual formation of Na_2_CO_3_ to the detriment of Na_2_CO_3_·H_2_O must be considered; as a result, water is released into the liquid bulk and it counterbalances the further addition of NaOH. In fact, when all the sodium into the system is converted to Na_2_CO_3_ and no more H_2_O is released by the dissolution of Na_2_CO_3_·H_2_O, the activity of water rapidly drops down to just above 0.400 at NaOH 20 m. That corresponds to a higher water activity aH_2_O within the liquid phase of the system (Fig. 8B), and therefore resulting in a higher proportioning of Na_2_CO_3_·H_2_O with respect to Na_2_CO_3_ (Fig. 8A).

### 4.3 Calculation of *E*_a_

**Fig. 9:**
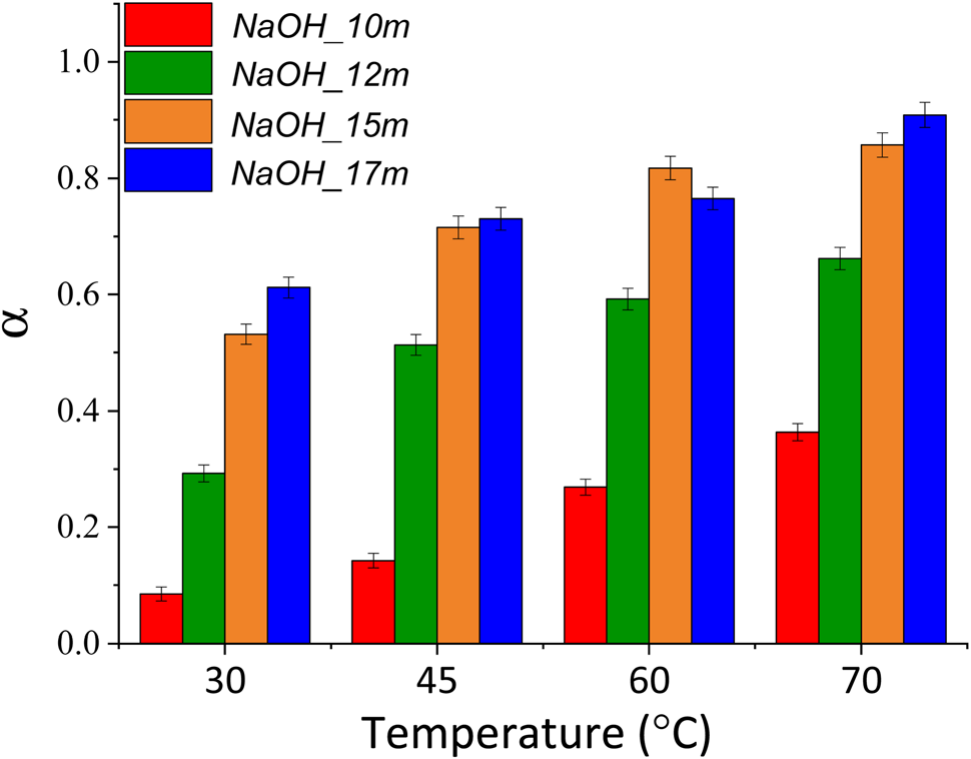
Conversion extent values (*α*) of the samples NaOH_10m_n, NaOH_12m_n, NaOH_15m_n and NaOH_17m_n plotted against the temperature initially set up and kept constant throughout the reaction.

An additional set of experiments was conducted to estimate the activation energy *E*_a_ of the decarbonisation reaction at the different starting compositions reported in Table 1, selected from our previous study.^16^ Based on the amount of CaCO_3_ and Ca(OH)_2_ in the reaction products estimated from their TGA data (ESI I–IV†), the extent of reaction (*α*) was calculated for each reaction. As reported in Fig. 9, beneficial effects of raising temperatures were observed at different levels depending on the starting composition.

In these terms, the effects of temperature (*T*) is commonly expressed in the reaction rate constant (*k*) using Arrhenius equation (eqn (7)),^38^ depending on the pre-exponential factor (A), activation energy (*E*_a_) and gas constant (*R*).

7 *k* = *A* e^(−*E*_a_/*RT*)^

The rate constant *k* is a change in the extent of reaction (*α*) per unit time (*t*). Since the reactions in this sub-set of experiments were all conducted for 1 min, the extent of reaction obtained (Fig. 9) was directly used in eqn (7) as representative of the reaction rate, and thus:

8 *α* = *A* e^(−*E*_a_/*RT*)^

Taking the natural logarithm, eqn (8) can also be expressed as:9
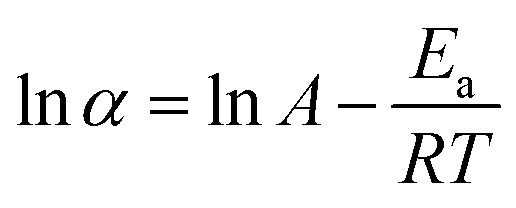


**Fig. 10:**
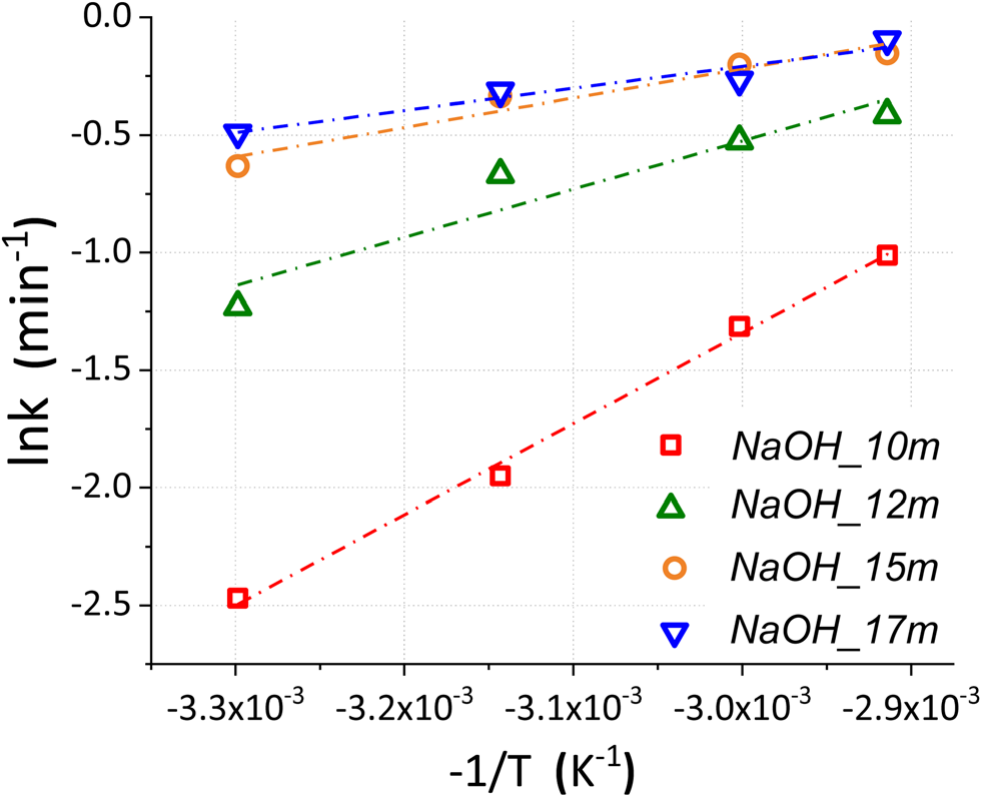
Linearized Arrhenius plot showing the correlation between ln *k* and −1/*T* (K^−1^) for the samples series NaOH_10m_n, NaOH_12m_n, NaOH_15m_n and NaOH_17m_n, reflected by the equations, 9, 10, 11, and 12, respectively.

By plotting ln *α* against *T*^−1^ (Fig. 10), the activation energy *E*_a_ can be calculated from the slope (−*E*_a_/*RT*); the experimental data for NaOH_10m, NaOH_12m, NaOH_15m, and NaOH_17m led to the fitting eqn (10)–(13), respectively.

10 *y* = 3865.1*x* + 10.254 *R*^2^ = 1.00

11 *y* = 2054.3*x* + 5.638 *R*^2^ = 0.91

12 *y* = 1242.1*x* + 3.507 *R*^2^ = 0.94

13 *y* = 933.3*x* + 2.591 *R*^2^ = 0.93

Based on these, the apparent activation energies for NaOH_10m, NaOH_12m, NaOH_15m and NaOH_17m are estimated as 32.1, 17.1, 10.3 and 7.8 kJ mol^−1^, respectively, and therefore reflecting the limited kinetics at lower NaOH molality. Despite that, lower activation energies were highlighted here, with respect to the conventional calcination route of CaCO_3_ under inert atmospheres (164–225 kJ mol^−1^^39^) and with varying CO_2_ partial pressures (213.3–2142.2 kJ mol^−1^^40^). Naturally, this first comparison does not consider the embodied energy required to produce the required NaOH to carry out the chemical calcination of CaCO_3_. For this reason, the next section will introduce crucial aspects to fully consider when assessing its real feasibility from an industrial point of view.

## 5 Industry-oriented considerations

The authors understand that a proper life-cycle assessment needs to be done to understand the industrial feasibility of this novel chemical decarbonisation process, but this is not the aim of the present investigation. Nevertheless, some crucial considerations are reported below to better highlight the potential of the decarbonisation route presented here.

It is highlighted that eventual fluctuations of temperature would not negatively affect the reaction yield; moreover, the mild processing conditions required suggest convenient operating costs. However, the energy input required for the synthesis of the stoichiometric NaOH would represent the major obstacle in sight of an industrial application. In fact, 7.7 GJ is required to produce 1 t of NaOH, which occurs alongside the synthesis of 0.03 t of hydrogen gas and 1.1 t of chlorine gas, through the chlor-alkali process.^41^ Such hydrogen is typically wasted and vented to the atmosphere, whereas the *in situ* recirculation for power production would lower the energy demand by approximately 34%.^42^ In these conditions, considering that 0.8 t of stoichiometric NaOH are required for each tonne of CaCO_3_ reacted, about 3.9 GJ_Equivalent_ of electricity should be supplied through usage of fresh fuel. Whereas, the conventional cement production route requires an energy input of 2.5–2.9 GJ per tonne of CaCO_3_ to decarbonise, considering the theoretical heat of dissociation of CaCO_3_ (1819.4 kJ^43^) and efficiencies between 41 and 62%.^44^ At first glance, our route does not appear economically desirable, since it requires 1.4–1.6 times the energy involved in the conventional route. However, it must be considered that the co-production of Na_2_CO_3_ occurs whilst synthetising Ca(OH)_2_, and important energetic considerations would arise from that. In fact, the production of 1 tonne of Na_2_CO_3_ requires 13.6 GJ through the conventional Solvay process,^45^ which would be completely avoided by supplying the product *via* the chemical route proposed. According to the stoichiometry depicted in eqn (1), a Na_2_CO_3_/Ca(OH)_2_ weight ratio of 1.43 occurs at the end of the reaction, assuming total conversion of CaCO_3_. The following considerations are done by normalising all the calculations with respect to cement, lime, and slaked lime, treating Na_2_CO_3_ as an added-value by-product. For the calculations, a 1 : 1 weight ratio between the decarbonised CaCO_3_ and the resulting cement was considered; in fact, the CaO proportion in PC is around 60–70%,^46^ and a 0.56 weight ratio occurs between the produced CaO (upon dehydration of Ca(OH)_2_) and the reacted CaCO_3_. For clarity, these numbers are reported in Table 3, alongside the cases for lime and slaked lime productions.

**Table 3:** Amounts of NaOH reacted and Na_2_CO_3_ produced through the chemical route by considering the stoichiometry depicted in eqn (1), together with the amount of CaCO_3_ required for both the thermal and chemical approach. The values reported refer to the normalisation of the products, case by case, PC, Lime and Slaked lime

Production type	PC	Lime	Slaked lime	CaCO_3_ reacted	NaOH reacted	Na_2_CO_3_ produced
PC	1	—	—	1	0.8	1.0
Lime	—	1	—	1.6	1.3	1.8
Slaked lime	—	—	1	1.2	1.0	1.4

**Fig. 11:**
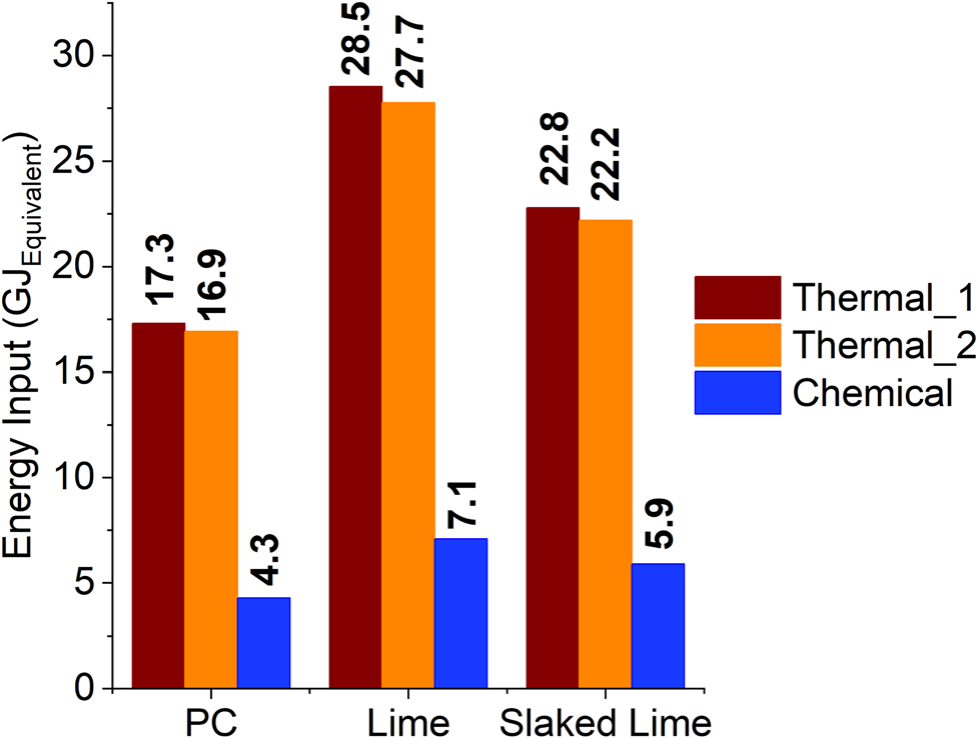
Comparison of the energy consumption between the thermal and chemical approaches to the decarbonisation of CaCO_3_, referred to the cement, lime and slaked lime industries; the values only depend on the decarbonisation step, considering a PRK design for the thermal calcination in PC production, and PFRK one for lime and slaked lime.

All these considerations were used to produce the energetic comparisons displayed in Fig. 11, where the electrical energy input required to produce the stoichiometric NaOH is reported alongside the thermal energy to be supplied to decarbonise CaCO_3_ plus the one to produce soda ash. The extremes of the energy efficiencies reported for the PRK design are labelled as “Thermal_1” (*η* = 41%) and “Thermal_2” (*η* = 62%) for the PC case, whereas the novel decarbonisation route is displayed as “Chemical” both for PC, lime, and slaked lime. It must be mentioned that the energies outlined were normalised with respect to 1 tonne of PC, according to the stoichiometry just discussed, and therefore expressed in GJ_Equivalent_, *i.e.*, the energy required to produce 1 tonne of PC and the stoichiometric amount of Na_2_CO_3_ (1.0 t). It must be mentioned that these values solely refer to the decarbonisation step, without considering additional consumptions associated with the further processing of both reactants and products. It is revealed that chemical decarbonisation allows for ∼4 times lower energy consumption with respect to the thermal route, considering the synthesis of 1.0 tonne of PC and 1.0 tonnes of Na_2_CO_3_ (Table 3). For the calculation of the energy requirement from the chemical route, a 10% surplus was considered to include the handling and separation of the materials.

Regarding the production of lime, 1.6 tonnes of initial CaCO_3_ are required to ensure 1 tonne of product, corresponding to a higher consumption of NaOH with respect to PC. For this reason, the normalisation with respect to lime shows that a higher amount of soda ash is produced, and that is considered for the calculation of the thermal power required from the conventional route. In these terms, the calcination design adopted in the lime/slaked lime industry allows for higher efficiencies (75% < *η* < 99%^44^); in Fig. 11, the labels “Thermal_1” and “Thermal_2” refer to the efficiency extremes of 75 and 99%, respectively. The larger consumption of NaOH reflects a higher energy input for the chemical route with respect to PC; despite that, even in this case the thermal route would require ∼4 times the energy employed in the chemical decarbonisation, when 1 and 1.8 tonnes of CaO and Na_2_CO_3_ are produced, respectively (Table 3). Finally, the case study for slaked lime Ca(OH)_2_ highlighted a 3.7–3.8 times lower energy input for the chemical route with respect to the conventional one for the synthesis of 1 and 1.4 tonnes of slaked lime and soda ash, respectively.

The preliminary energetic balance appears promising when sodium carbonate is considered as a co-product (*i.e.*, replacing market soda ash); however, the global market demand of the chemicals involved must be considered. In fact, the current global demand for PC and soda ash is 4 Gt^1^ and 50 Mt,^47^ respectively; if the whole global production of PC is performed chemically, an excess production of soda ash would occur. However, the demand for soda ash might significantly grow given the increasing importance of geopolymers in the global perspective, with Na_2_CO_3_ used as activator to produce these low-carbon binders.^48^ Furthermore, in case the excess of soda ash could not be reused, Na_2_CO_3_ would represent a safer option for permanent CO_2_ storage with respect to the current geological disposal of liquid and compressed CO_2_, whose long-term effects have not been fully understood yet.^49^

Another drawback of the chemical route is represented by the production of chlorine gas arising from the chlor-alkali process to produce NaOH;^41^ if the size of the market increases to fulfil the demand for cement production, such emissions might have a heavy impact on the environment. The development of chlorine-based binders, such as alinite,^50^ could help to mitigate the issue. In fact, despite that the presence of Cl leads to the failure of reinforced concrete through corrosion of the mild steel,^51^ ∼75% of the worldwide cement is used for unreinforced purposes.^52^ In addition, an enhancement of the chlor-alkali process with the *in situ* hydrogen processing and re-use would contribute to the mitigation of the drinkable water crisis expected in the next decades.^53^ In fact, Na^+^ and Cl^−^ are removed from the brine (concentrated seawater) fed into the chlor-alkali process, and pure water is a by-product from the reprocessing of hydrogen.^54^

Alternatively, both the excess production of soda ash and the increasing emissions of chlorine gas might be limited by solely applying the novel route to produce lime and slaked lime, whose markets (50 Mt and 20 Mt, respectively^2^) are currently closer to that of soda ash (50 Mt^47^).

## 6 Conclusions

The effects of temperature on the efficiency of a chemical decarbonisation process for CaCO_3_ was assessed. The reaction appeared effective for the sequestration of the process CO_2_ partitioned into CaCO_3_, with over the 80% of it captured within the first minute of reaction. The short residence times and mild heating conditions required look promising in sight of an eventual scale-up. It is supposed that the process might be thermally self-sustained, given (1) the exothermic dissolution of NaOH into water, and (2) the high conversion degrees observed even at ambient conditions. That would reflect a significant advantage from an industrial point of view, suggesting that the process is robust and that no additional costs would be associated with the control of the reaction temperature.

The capture of the process CO_2_ occurs through precipitation of Na_2_CO_3_·H_2_O at milder temperature and pH, whereas Na_2_CO_3_ is the dominant Na-based species at higher temperature and alkalinity. Given the need for a final separation of Ca(OH)_2_ from Na_2_CO_3_. xH_2_O, not discussed here, Na_2_CO_3_·H_2_O might be favourable given the higher solubility with respect to Na_2_CO_3_ (330 and 307 g L^−1^ at 25 °C and 1 atm, respectively). In fact, given the much lower solubility of Ca(OH)_2_ (1.5 g L^−1^ at 25 °C and 1 atm), the separation can be carried out in excess of water, and the yield would be maximised when the solubility gap between the phases of interest is large.

The activation energy *E*_a_ was determined for different ternary compositions previously tested. Generally, the activation energy barrier was smaller for the system with the higher initial NaOH concentration; moreover, the rate of the chemical decarbonisation appeared much higher with respect to the conventional CaCO_3_ calcination for all the conditions tested.

Preliminary energetic considerations were also reported, and the comparison between the thermal and chemical route to produce PC, lime, slaked lime, and soda ash was shown. As outlined, the chemical route was ∼4 times more convenient in terms of energy input, if only the decarbonisation step of CaCO_3_ is considered and when soda ash is considered as a co-product.

Overall, the chemical decarbonisation of CaCO_3_ may have the potential to drastically reduce the carbon footprint of the related industries, potentially removing the need for high temperature calcination. The main obstacle to overcome is still represented by the general concept that the thermal decarbonisation of CaCO_3_ is unavoidable, and that a transition would be technically too difficult to operate. However, the current environmental crisis demands for brave and optimistic approaches/decisions backed by the desire for real change towards a sustainable future.

## Author contributions

Theodore Hanein and Hajime Kinoshita discovered and conceptualized the technology. Marco Simoni developed the technology and designed the methodology and experiments. Marco Simoni and Chun Long Woo carried out experiments. Marco Simoni drafted the original manuscript. Theodore Hanein, Hajime Kinoshita, and John L. Provis acquired funding, and supervised Marco Simoni and Chun Long Woo. Theodore Hanein, Hajime Kinoshita, John Provis, Mark Tyrer, and Magnus Nyberg reviewed and edited the manuscript.

## Conflicts of interest

The authors declare that they have no competing interests as defined by RSC Advances, or other interests that might be perceived to influence the interpretation of the article.

## Supplementary Material

RA-012-D2RA05827H-s001
