# Supplementary material for: Synthesis of Ca(OH)2 and Na2CO3 through anion exchange between CaCO3 and NaOH: effect of reaction temperature
Source: RSC Adv. 2022 Nov 11;12(49):32070–81. doi: 10.1039/d2ra05827h (PMC9650501; doi:10.1039/d2ra05827h)
Supplement: RA-012-D2RA05827H-s001 [file RA-012-D2RA05827H-s001.pdf]

Electronic Supplementary Material (ESI) for RSC Advances.
This journal is © The Royal Society of Chemistry 2022

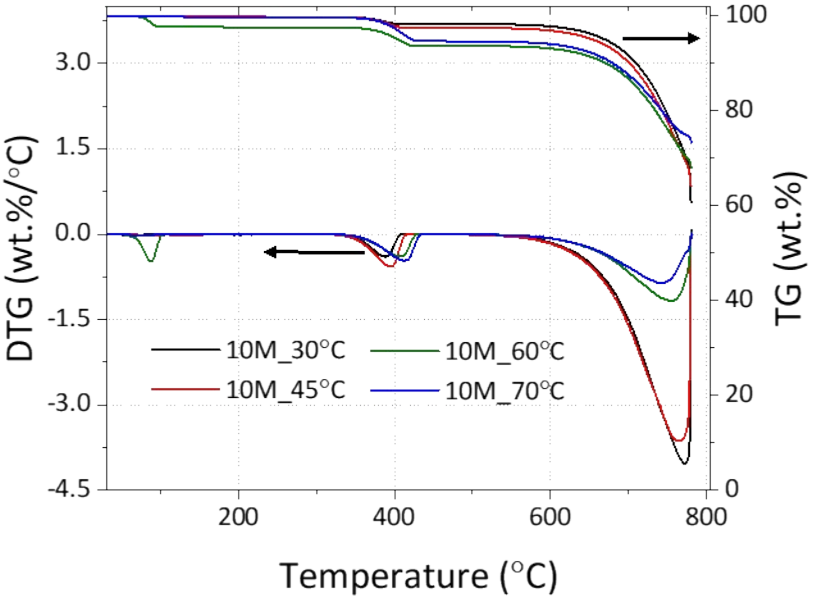

SEI_I: TG/DTG trends registered for the samples reacted at 30, 45, 60, and 70°C in 10M NaOH solutions.

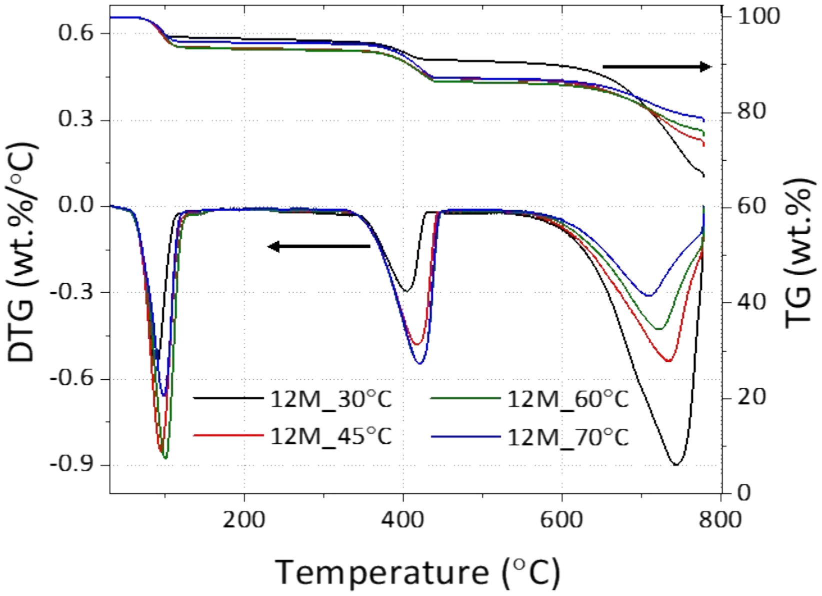

SEI_II: TG/DTG trends registered for the samples reacted at 30, 45, 60, and 70°C in 12M NaOH solutions.

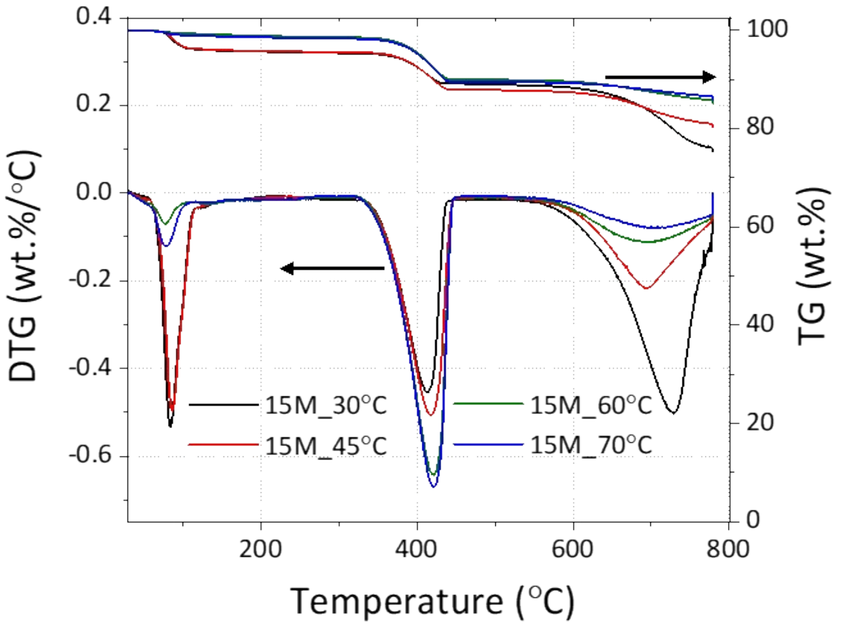

SEI_III: TG/DTG trends registered for the samples reacted at 30, 45, 60, and 70°C in 15M NaOH solutions.

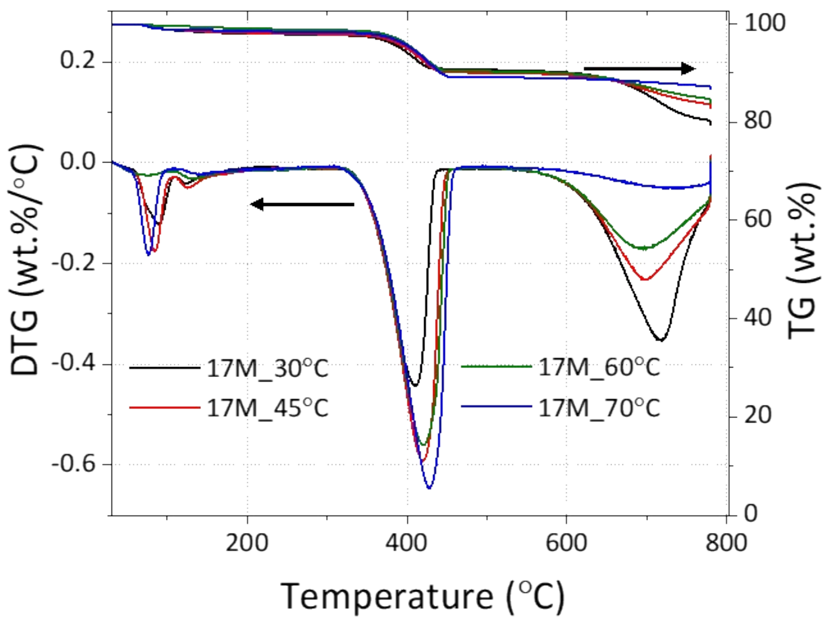

SEI_IV: TG/DTG trends registered for the samples reacted at 30, 45, 60, and 70°C in 17M NaOH solutions.
